# A case report of traumatic osteoarthritis associated with LARS artificial ligament use in anterior cruciate ligament reconstruction

**DOI:** 10.1186/s12891-020-03764-7

**Published:** 2020-11-12

**Authors:** Yuanliang Du, Haifeng Dai, Zhihui Wang, Di Wu, Changjiang Shi, Tianjie Xiao, Zhihuai Li

**Affiliations:** Department of Joint Surgery, Affiliated Hospital of Chengde Medical University, No.36 Nanyingzi Street, Chengde, Heibei Province China

**Keywords:** Anterior cruciate ligament, ACL, Ligament advanced reinforcement system, LARS, Osteoarthritis, Ligament reconstruction

## Abstract

**Background:**

A ligament advanced reinforcement system (LARS) artificial ligament has been proposed for use in anterior cruciate ligament (ACL) reconstruction, and many reports have shown its success in ACL reconstruction. However, there are great concerns about the potential risk of complications, which might prevent its extensive use. Late failure may occur due to serious complications.

**Case presentation:**

We report a rare case of serious osteoarthritis that occurred 2 years postoperatively in a 51-year-old man who underwent reconstruction with an LARS artificial ligament. In X-rays, the tibial tunnel was placed too posteriorly. MRI showed that the tibial tunnel was enlarged, and there was a large effusion in the knee joint. The LARS device was rough and worn. Histologically, a large number of fibroblasts and a few multinucleated giant cells infiltrated the graft fibres.

**Conclusion:**

Our findings remind surgeons that an LARS device should be with great caution in ACL reconstruction.

## Background

Anterior cruciate ligament (ACL) injury is the most common reason for knee instability, and injuries to the ligament may lead to osteoarthritis and other degenerative joint disease [1, 2]. For patients eager to achieve orthobiosis, the most effective treatments for ACL injury are reconstruction of the ligament and postoperative physiotherapeutic procedures [3]. Reconstruction of the ligament could be conducive to knee stability and, in turn, reduce the risk of secondary injuries [4, 5]. A variety of grafts are available for use in ACL reconstruction surgery, such as allografts and synthetic ligaments. The use of allograft autografts are the first option in ACL-reconstruction in recent years with the attraction of absent donor site morbidity [6]. The ligament advanced reinforcement system (LARS), a synthetic, non-absorbable augmentation device made of polyethylene terephthalate (PET), has been also widely used in many countries [7]. With the aim of removing potential machining residues and oils, the LARS is intensively cleaned to further encourage soft tissue in-growth and reduce the risk of reactive synovitis [8]. In a recent study, the medium-term clinical outcomes of ACL reconstruction using an LARS artificial ligament were assessed, and among all patients enrolled in the study, the failure rate was 4.4%, with an overall complication rate of 2.2% [9]. Although these preliminary clinical investigations have shown encouraging results, there is concern that late failure may occur due to serious complications. We present a case report of osteoarthritis after LARS artificial ligament implantation that required revision at 2 years and a histologic analysis of the retrieved artificial ligament. This case highlights the seriousness of potential hazards when using an LARS artificial ligament, and further research may focus on its long-term outcomes after ACL reconstruction.

## Case presentation

**Fig. 1:**
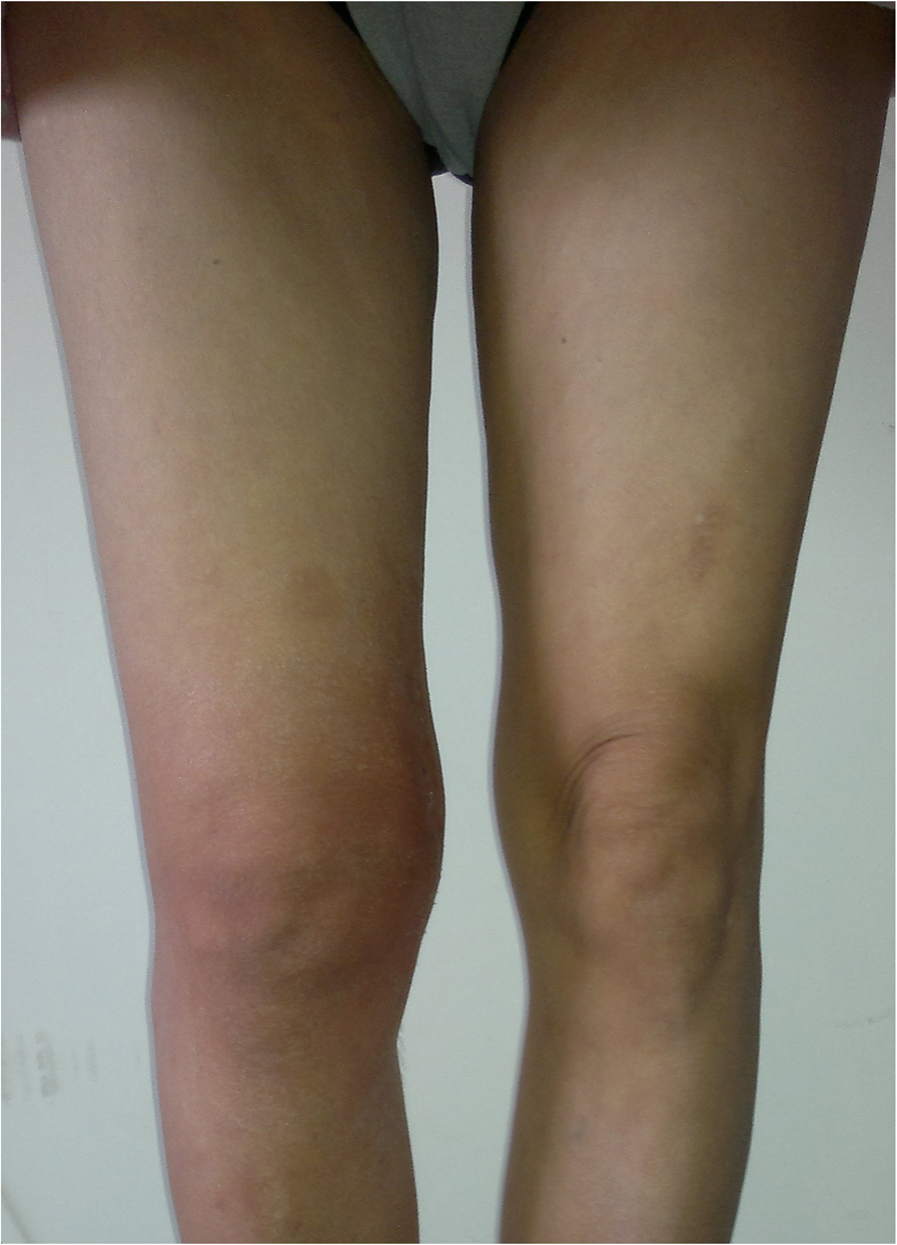
The right knee joint was obviously swollen in the standing position

**Fig. 2:**
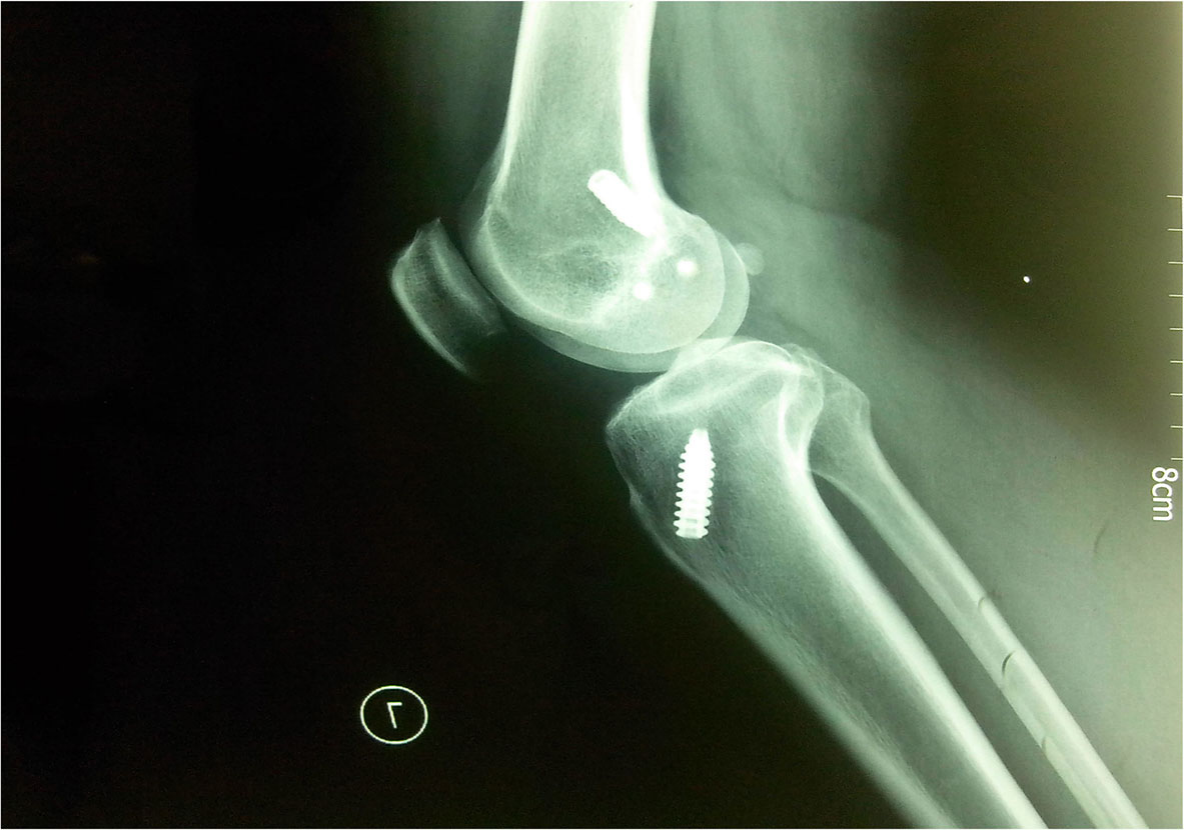
Preoperative X-rays demonstrated femoral and tibial tunnel placement in a lateral view

**Fig. 3:**
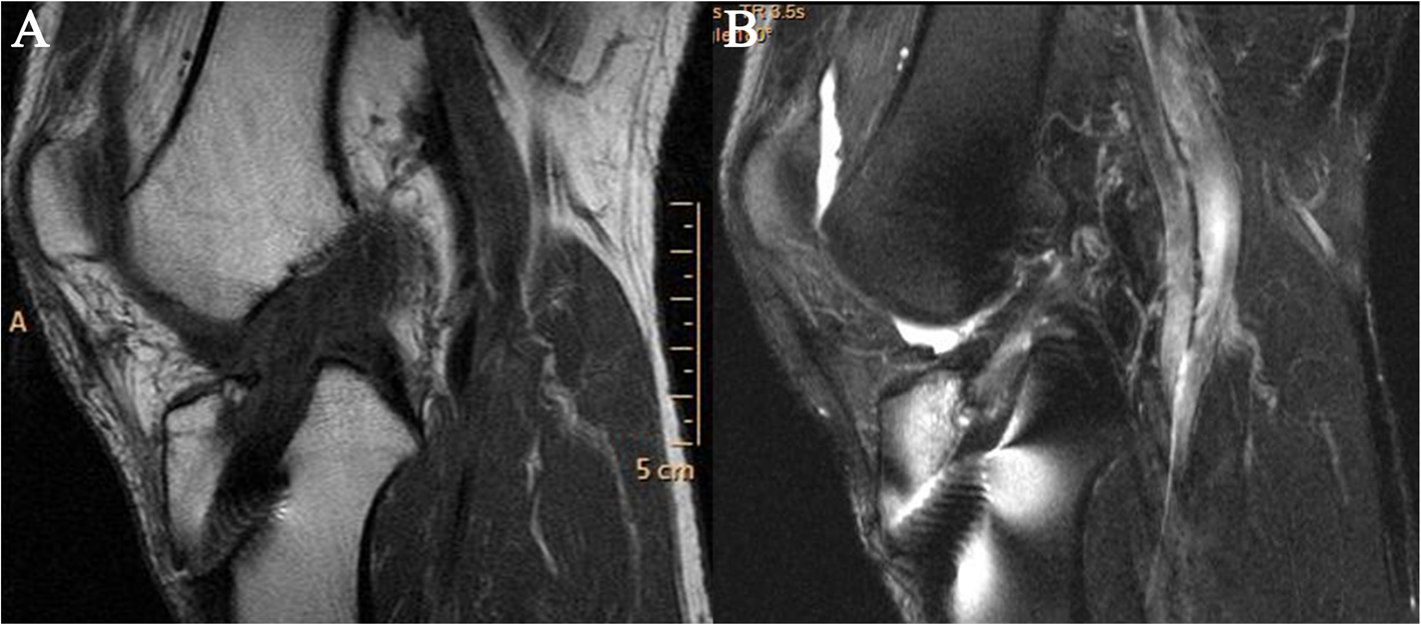
Preoperative MRI showed that the tibial tunnel was enlarged on the T1 sequence (**a**); on the T2-weighted images, there was a large effusion on both the femoral and tibial sides (**b**)

A 51-year-old worker sustained serious knee synovitis and osteoarthritis after ACL reconstruction with an LARS ligament 2 years previously. Knee arthroscopy was performed, and the ruptured artificial ligament was retrieved for histologic study. Subsequently, the patient underwent prosthetic replacement of the knee joint. Originally, the patient underwent ACL reconstruction with an LARS artificial ligament in the right knee for an ACL injury in 2011 in our department. After surgery, the postoperative physiotherapeutic procedure was followed. Postoperative recovery consisted of non-weightbearing on crutches in a hinged brace with a progressive increase in range of motion. The brace was removed 8 weeks after surgery, and full weightbearing was allowed to commence at 12 weeks. At 9 months, the patient reported continual aching in the knee joint after a long walk. No extra treatment was started except for more rest, and physical therapy was resumed. However, the patient had sustained, repeated pain and swelling in the right knee since then. The patient could not walk and suffered from continual aching, which could not be relieved after rest, 1 year later. During the initial physical examination, the patella tap test, anterior drawer test, and Lachman test were all positive. The right knee joint was obviously swollen before surgery (Fig. 1). X-rays demonstrated that the tibial tunnel was placed slightly posteriorly (Fig. 2). Magnetic resonance imaging showed that the tibial tunnel was enlarged on the T1 sequence, which led to loss of stability and chronic inflammation (Fig. 3a). On the T2-weighted images, there was high signal intensity on both the femoral and tibial sides, and there was an effusion in the joint (Fig. 3b). Internal fixation removal of the LARS artificial ligament and prosthetic replacement of the knee joint were recommended.

### Surgery

**Fig. 4:**
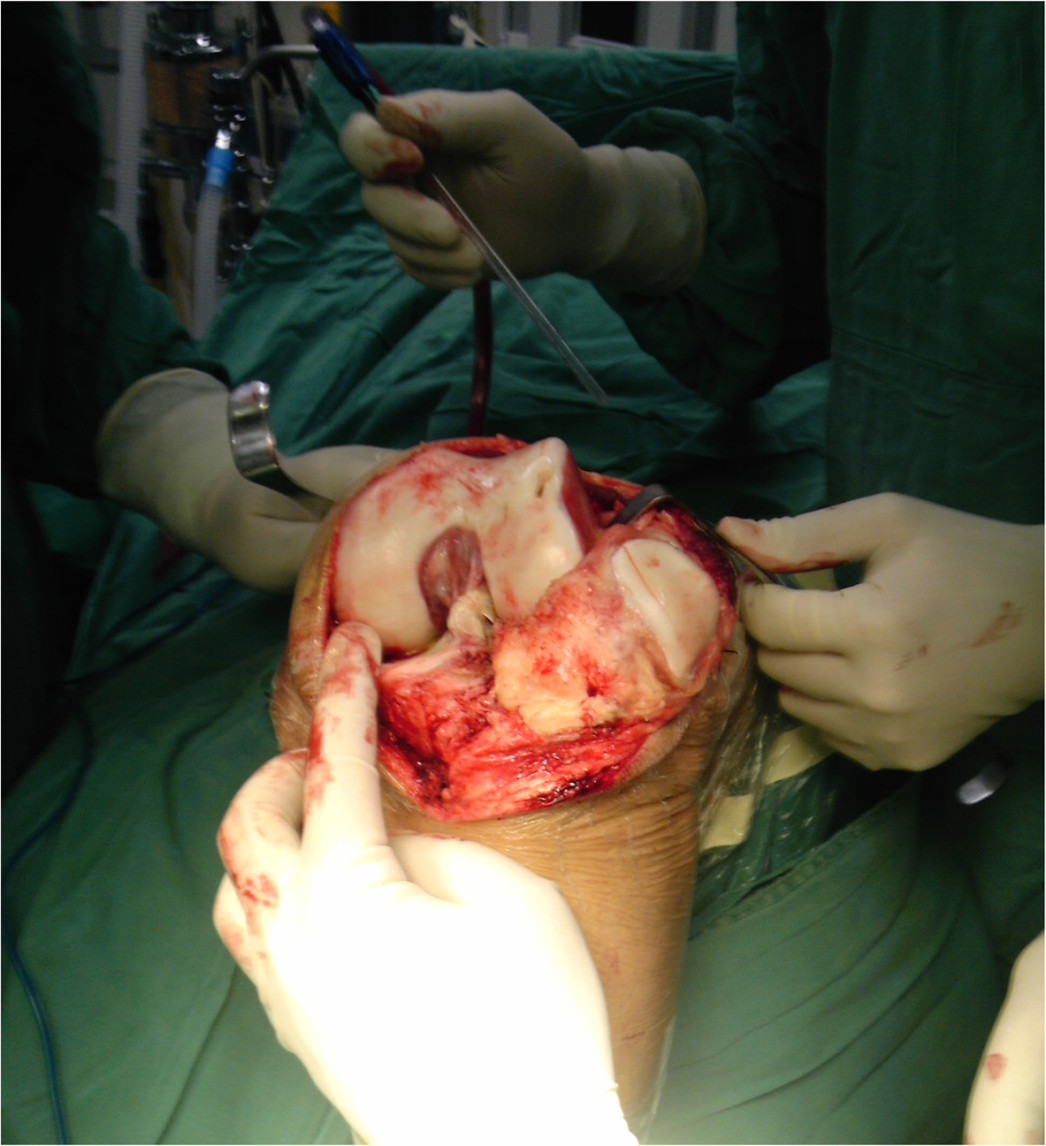
Intraoperative photographs showed severe widespread synovitis in the knee joint

**Fig. 5:**
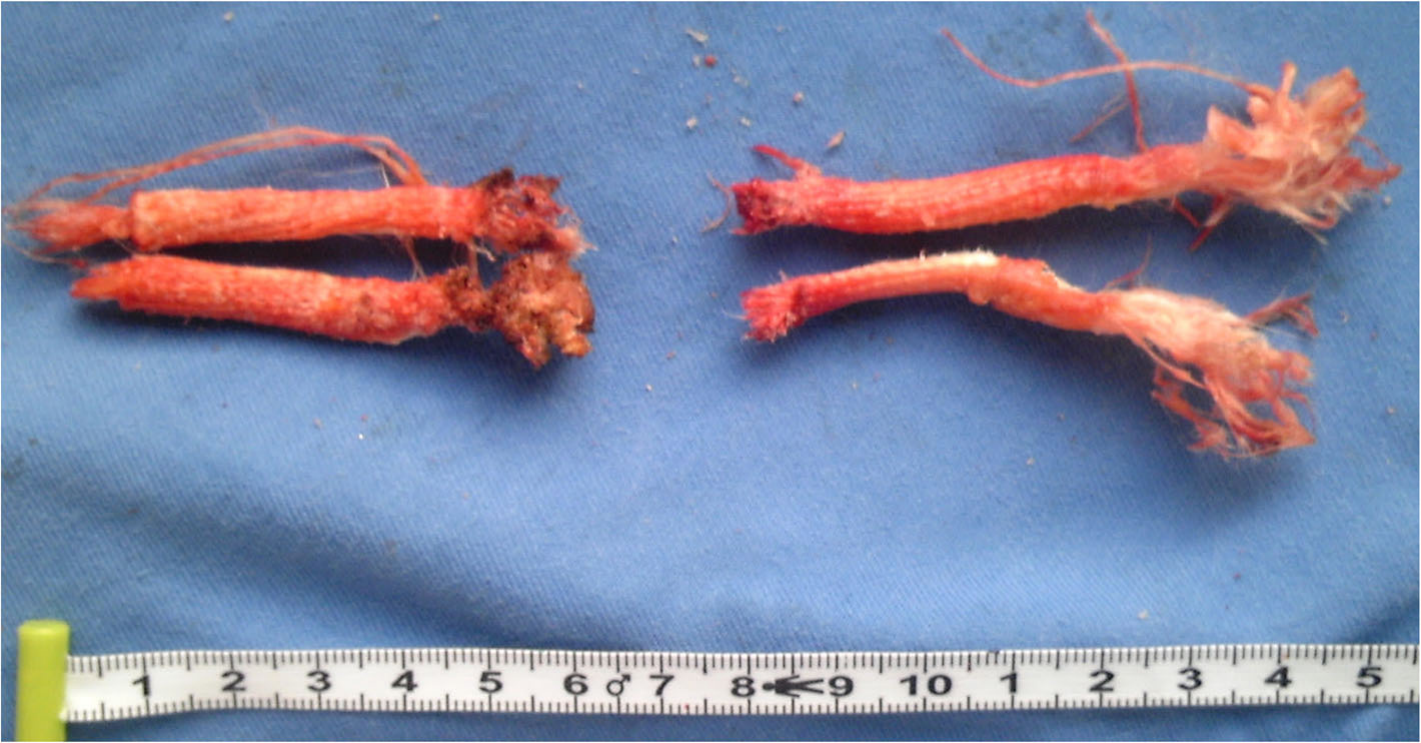
The LARS device was rough and worn with visible polyethylene terephthalate debris

First, the skin and subcutaneous tissue were cut, freeing approximately 3 cm on both sides along the deep subfascial space and exposing the front of the knee joint and the quadriceps tendon. The suprapatellar capsule and joint capsule were cut along the quadriceps tendon and the medial edge of the patella to the inside of the tibial tubercle. The effusion was drained, the tibia was evaginated, and the knee joint was flexed. Villous synovitis was evident in all compartments, and the medial tibial plateau, medial femur and patellar cartilage were all worn (Fig. 4). The intercondylar notch was narrow, and the meniscus had metamorphosed. The tibial tunnel was placed too posteriorly, and the LARS device was rough, worn and incompletely enshrouded in fibrous tissue (Fig. 5). The intra-articular LARS prosthesis was excised to reveal the titanium femoral interference screw, which was removed completely by special equipment. Simultaneously, the femoral and tibial ends of the prosthesis were able to be removed. After that, the femoral condyle, meniscus and tibia were replaced by artificial joint prostheses.

### Histopathological analysis

**Fig. 6:**
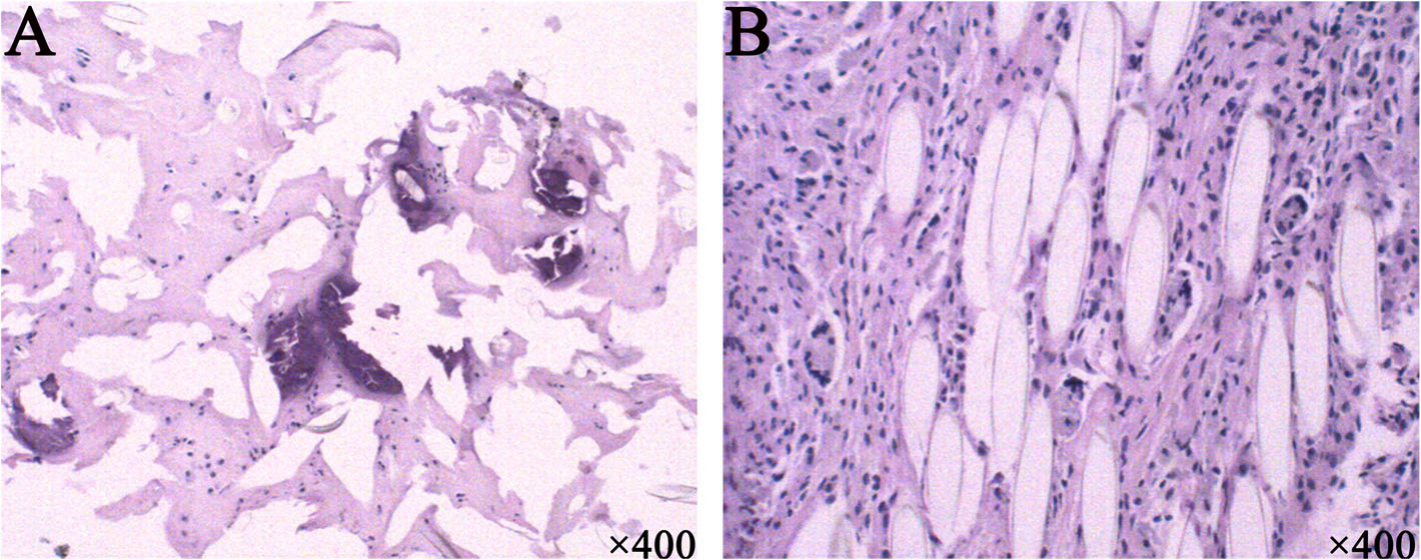
Histologic examination showed that proliferated chondrocytes can be seen around the artificial ligament with calcification in some areas (**a**). A large number of fibroblasts and a few multinucleated giant cells around the artificial ligament were observed between the fibrous tissues (**b**). The magnification was × 400

The excised LARS prosthesis was divided into twelve segments to perform histopathological analysis. On light microscopy, marked hyperplasia was observed around all parts of the graft, with many multinucleate cells surrounding and inside the graft fibres (Fig. 6a). Approaching the tibial end of the graft, there were large amounts of proliferating chondrocytes inside the lacuna, along with some visible calcification (Fig. 6b). The closer the graft was to the tibia, the more obvious the phenomenon. However, only fibroblasts and poorly multinucleated cells infiltrated into the artificial ligament in the femoral end without any chondrocytes. There was no evidence of malignancy. The findings were consistent with potential involvement of the LARS ligament, which allows new ligamentous and neurovascular tissue to regenerate along a synthetic scaffold.

## Discussion and conclusion

Theoretically, LARS artificial ligaments made of terephthalic polyethylene polyester fibres are a great innovation. The aims of its design are to prevent fibre breakdown early and facilitate tensioning of the graft fibres during knee movement. In contrast to traditional ACL reconstruction techniques, the LARS surgical technique reduces trauma to the soft tissues of the knee with less surgical time [10, 11]. Although the LARS has made achievements in the short term, serious complications have been reported, such as graft rupture, foreign-body inflammation, and serious knee synovitis [12–14].

In one study that evaluated the clinical outcome of ACL reconstruction with LARS with at least a 7-year follow-up, the failure rate was only 4.4%, including two cases of recurrent instability, one case of limitation of the range of motion and one artificial graft rupture; however, no synovitis occurred in the study [9]. In a multicentre study with 3 to 5 years of follow-up in 159 patients, the overall complication rate for ACL reconstruction with LARS artificial ligaments was 5.7%, and serious knee synovitis developed only in one case [15]. Although synovitis appears to be a rare complication, it is very serious and can result in ligament rupture and surgical failure [16–18]. In addition, synthetic grafts in ruptured ACL were associated with a lack of subjective satisfaction in half of patients treated with LARS [19]. The most common cause of complications was closely related to imperfect graft positioning: either the tibial or femoral tunnel was too far anteriorly or both [20, 21]. Amis and Kempson examined 25 ruptured polyethylene terephthalate fibre ACL implants retrieved clinically and believed that ACL implant failure was often caused by bone impingement during knee extension after malpositioning of the tibial tunnel (often too far anteriorly) [22]. However, in our case, the tibial tunnel was placed slightly posteriorly, and the ligament was ruptured near the tibial tunnel. The tunnel positioning in this case might have led to knee joint laxity and loss of stability, causing signs of osteoarthritis. With osteoarthritis in the knee joint, in turn, the LARS ligament could not achieve adequate tensile strength to maintain joint stability, which accelerated the process of ligament rupture. Therefore, non-anatomical reconstruction is the direct reason for surgical failure.

In this case, we left sufficient intact tissue to completely enshroud the LARS ligament in the femoral stump, intending to encourage fibrovascular ingrowth around individual fibres and hence prevent the articular release of wear particles. Based on observation of the histological analysis, only a few chondrocytes grew well along with the parallel fibres of the LARS ligament. However, the ligament should be highly cleaned to remove potential machining residues and oils to further reduce the risk of reactive synovitis and osteoarthritis. While coverage may theoretically prevent wear particles from entering the joint, the native residues or tissue remains shielded from stress by the nonabsorbable LARS graft, and when it does fail, the tissue shielded from stress will also fail, resulting in laxity and exposure of the joint to wear debris.

Although it would be unwise to draw too firm a conclusion from one case, the present case confirmed that LARS ligaments promote chondrocytes proliferation in terephthalic polyethylene polyester fibres with enough native residues by histologically analysis. However, the synovitis and osteoarthritis in this case might have resulted from shielded tissue failure. How to modify the residue surface requires further research to promote the biocompatibility of the LARS artificial ligament. Additionally, in the absence of peer-reviewed long-term outcome data, further use of the LARS device should be done with great caution.

## Data Availability

All data generated or analysed during this study are included in this published article.

## Abbreviations

ACL: Anterior cruciate ligament
LARS: Ligament advanced reinforcement system
